# Reduced Volumetric Bone Mineral Density of the Spine in Adolescent Rett Girls with Scoliosis

**DOI:** 10.3390/children9121902

**Published:** 2022-12-04

**Authors:** Konstantinos Tsaknakis, Jan H. Kreuzer, Friederike Luise Metzger, Katharina Jäckle, Katja A. Lüders, Lena Braunschweig, Heiko M. Lorenz, Anna K. Hell

**Affiliations:** Paediatric Orthopaedics, Department of Trauma, Orthopaedic and Plastic Surgery, University Medical Center, 37077 Göttingen, Germany

**Keywords:** Rett syndrome, vBMD, volumetric bone mineral density, scoliosis, Z-score, valproate

## Abstract

In advanced Rett syndrome (RTT), limited or complete loss of ambulation, nutritional problems and scoliosis are unfavorable factors for bone mineral density (BMD). Still, there are few data available in this research area. Spinal quantitative computed tomography (QCT) allows an exact measurement of the volumetric BMD (vBMD) in this patient group. Two examiners measured vBMD of thoracic and lumbar vertebrae on asynchronous calibrated CTs that were acquired prior to surgical scoliosis correction (n = 21, age 13.6 ± 2.5 years). The values were compared to age- and sex-matched healthy controls to additionally derive Z-scores (n = 22, age 13.8 ± 2.0 years). The results showed the most significant reduction of vBMD values in non-ambulatory RTT patients, with *p* < 0.001 and average BMD-Z-score −1.5 ± 0.2. In the subgroup comparison, non-ambulatory patients with valproate treatment had significant lower values (*p* < 0.001) than ambulatory patients without valproate therapy, with an average BMD-Z-score of −2.3 ± 0.2. Comparison of the Z-scores to critical BMD thresholds of 120 and 80 mg/cm^3^ showed normal Z-scores in case of the ambulatory RTT subgroup, as opposed to BMD-Z-scores of the non-ambulatory RTT subgroups, which were partially below osteopenia-equivalent values. Furthermore, valproate treatment seems to have a direct effect on vBMD in RTT patients and when combined with loss of ambulation, BMD-Z-scores are reduced to osteoporosis-equivalent levels or even further.

## 1. Introduction

Rett syndrome (RTT) [1,2] is an X-linked neurological disorder caused mainly through mutations in the methyl CpG binding protein 2 (MECP2) gene [3]. The affected girls demonstrate an early age stagnation followed by regression of cognitive and motor skills, epilepsy as well as characteristic behavioral patterns. This led to the first description of the syndrome in 1966 by Andreas Rett [4,5]. In 1985, Hanefeld described his observations on clinical symptoms of eight patients from early childhood to adulthood, which eventually led to the definition of four distinct development stages [6,7]. Further studies showed that disorders are already present at birth [8].

In the “early onset phase”, patients demonstrate developmental stagnation, followed by the “rapid destructive phase” during which girls lose learned skills. The “plateau phase” is a stable period, and lost skills may partially be reacquired and the typical repetitive hand movements appear. During the fourth “late motor deterioration phase”, girls develop neuromuscular dysfunction, reduced overall activity, known as apraxia, limited or complete loss of ambulation and spinal deformity progression. A multicenter study between 2006 and 2015 with 913 RTT patients showed a scoliosis prevalence of 27%. A surgical correction was performed in 168 cases [9].

**Figure 1:**
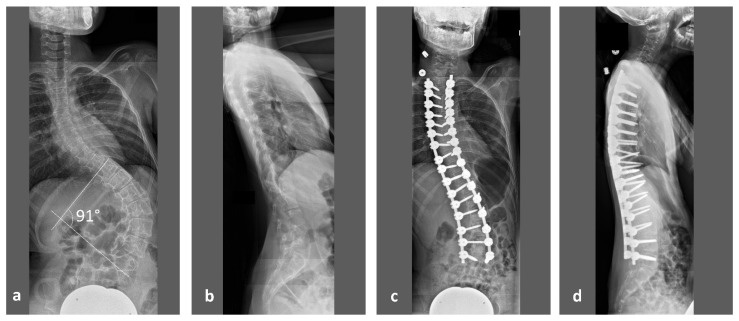
13-years-old, non-ambulatory girl with RTT with severe neuromuscular scoliosis and thoracic hyper-kyphosis. (**a**) Preoperative anteroposterior (ap) and (**b**) lateral sitting radiographs with a scoliosis angle of 91°. (**c**) Postoperative ap and (**d**) lateral sitting radiographs with dorsal spinal fusion from T (thoracic) 2 to L (lumbar) 5.

While early deformity during childhood is mainly treated by conservative bracing, advanced scoliosis will need surgical spinal deformity correction (Figure 1). In our clinic, preoperative computed tomography (CT) of the spine in sedation will be performed for deformity analysis, presurgical planning and intraoperative navigation. The same datasets also allow vertebral vBMD measurements through asynchronous calibrated quantitative computed tomography (QCT). Such measurements overcome inherent limitations of Dual-energy X-ray absorptiometry (DXA) when examining patients with disability. Stable positioning and severe scoliosis have been described by the International Society for Clinical Densitometry (ISCD) in its official pediatric positions as factors that may pose a problem for reliable DXA measurements [10]. Sedation of patients with mental disability during DXA can at least provide a solution for stable positioning [11]. However, it may prove difficult to justify such a screening examination when sedation for an inpatient is required. Studies on bone mass in RTT patients through DXA examinations have been described in literature which, while providing 2-dimentional areal BMD (aBMD) values [11,12], still do not overcome the problem of spinal deformity.

To our knowledge, for the first time, this study provides accurate results of vertebral vBMD in this patient population for the thoracic and lumbar spine. As the surgical correction of advanced neuromuscular scoliosis involves a fixation of practically all thoracic and lumbar vertebrae (Figure 1), these results may help to optimize results after operative treatment, to identify risk factors for osteoporosis and to improve conservative treatment regimens in RTT patients.

## 2. Materials and Methods

In this prospective cross-sectional cohort study, 21 girls (mean age 13.6 ± 2.5 years, range 11.0–20.6 years), who were diagnosed with RTT and progressive spinal deformity, which was not yet treated surgically, were included between 2017 and 2022. All participants were admitted to our clinic in preparation for definite spinal fusion to treat spinal deformity. The institutional ethical review committee of University Medical Center Göttingen approved our study proposal (reference number 33/8/17, approved 16 April 2017, and number 20/4/21, approved 27 April 2021) and the need for informed consent for this study was waived.

Demographic and clinical data were collected by interviewing the patients’ parents and from the medical records during the work-up, as well as follow-up examinations after surgery. Using Centricity Enterprise Web Version 3.0 (GE Healthcare Medical Systems, Chicago, IL, USA, 2006), scoliotic curves were measured on standardized standing or, if not possible, sitting anteroposterior (ap) as well as lateral radiographs.

Twenty-one asynchronous, phantom pre-calibrated, preoperative CT scans (Somatom Definition AS, Siemens, Erlangen, Germany) of the whole spine were analyzed for extraction of vBMD values of thoracic and lumbar vertebrae. All CT scans had a slice thickness of 0.6 mm. One girl had a pathological fracture of the femur in her medical history.

A cohort of 22 age-matched female patients (mean age 13.8 ± 2.0 years, range 10.6–17.9) undergoing a native CT (n = 14) or a contrast enhanced CT (n = 8) served as the control group. The CT was performed with the contrast agent Imeron^®^ 350 (Bracco Imaging Deutschland GmbH, Konstanz, Germany) with an iodine concentration of 350 mg/mL (n = 10) or with the contrast agent Ultravist^®^ (Bayer Vital GmbH, Germany, Leverkusen) with an iodine concentration of 370 mg/mL (n = 4) and a maximal slice thickness of 0.6 to 0.75 mm. The examined patients of the control group received CT examinations to rule out a pathology or to confirm conditions not relevant to BMD. These were high-energy trauma (n = 12), pectus excavatum (n = 3), lung examinations (n = 3), mediastinal examinations (n = 2), urinary tract examination (n = 1) and spondylolisthesis (n = 1). There were no vertebral fractures observable in any of the CT scans. Other verified conditions in the control group were one case of pulmonary embolism without underlying cause, one case of nephrolithiasis, one patella fracture, one case of atrial septal defect and one case of gastroesophageal reflux.

**Figure 2:**
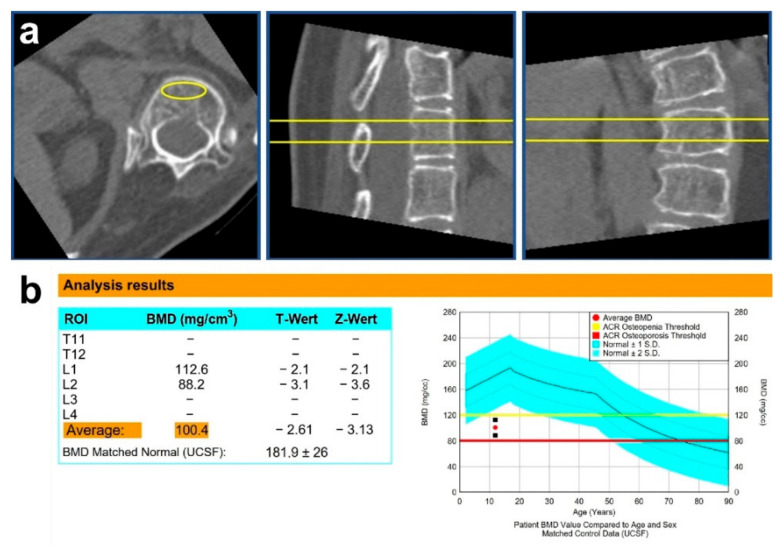
(**a**) The yellow marked region demonstrates the ROI (region of interest) in all three anatomical planes. Cortical bone and neurovascular tissue should not be inside this defined volume. (**b**) The vBMD results with age and sex matched Z-scores based on reference database of UCSF (University of California, San Francisco, CA, USA) [13,14,15,16,17]. The black squares represent the vBMD value of each vertebra and the red dot the average value.

The software QCTpro^®^ version 6.1 (Mindways Software Inc., Austin, TX, USA) was used to evaluate the CT data. A separate axial, sagittal, and coronal alignment for each thoracic and lumbar vertebra was necessary to compensate for spinal deformity and make the following determination of the range of interest (ROI) possible. The ROI defines the borders of the volume of the vertebra, which will be used to calculate the vBMD. This volume must lie in the vertebral body, including only cancellous bone, while excluding cortical bone and non-osseous structures, such as neurovascular tissue (Figure 2).

Because of advanced deformity of the spine, the ROI definitions were performed manually before extracting the BMD values. For this reason, measurements were carried out independently by two physicians to evaluate interobserver accuracy. Values with deviation of 5% or more from the mean were re-validated with additional measurements by a third examiner. Totally, 73 of 357 values per examiner (20.4%) had to be re-validated. Deviation of measurements were caused through non-trabecular structures in the ROI as described above. Furthermore, alignment of the anatomical planes was partly insufficient or the definition of the level of the evaluated segments was incorrect.

The CT scans of the control group could be evaluated by one examiner, because physiological vertebral anatomy and orientation allowed for a straightforward and almost automated process.

The vBMD values obtained through contrast enhanced CT scans (BMD_MDCT_) were converted (BMD_QCT_) according to the equation of Bauer et al. as described in 2007 to adjust for artificially higher measurements due to the contrast agent [18]:

BMD_QCT_ = 0.96 × BMD_MDCT_ − 20.9 mg/mL

Initially, this equation was conceived for the lumbar vertebrae L1 to L3, whereas in this study, measurements of all thoracic and lumbar vertebrae were performed. The statistical comparison of the converted vBMD values from the contrast enhanced CT scans to those of the native ones with an unpaired, two-tailed *t*-test showed no statistical difference (*p* < 0.92).

The Z-scores were calculated. These express the number of standard deviations (SD) of the average vBMD values of the RTT group compared to the average vBMD value of the control group for the same age [19]. The Z-scores are automatically generated by the software (Figure 2) but are derived from the reference vBMD values of the University of California, San Francisco (UCSF) database. According to the software documentation, these data are based on studies performed over forty years ago [13,14,15,16,17]. Therefore, control data were collected to calculate Z-scores, which were acquired on the same CT scanners as that of the RTT patients.

Height adjusted Z-scores (HAZ) to compensate growth are necessary when performing DXA examinations with areal BMD (aBMD), as taller persons also have vertebrae with larger diameter, thus leading to cumulative higher aBMD values [20]. In QCT-based vBMD, adjustments for height are not needed, as all three dimensions are already included in the calculations.

Thoracic and lumbar vertebral BMD values of ambulatory and non-ambulatory RTT patients were compared to the control group. The calculated Z-scores were compared to the threshold Z-scores, which correspond to the critical BMD values of 120 mg/cm^3^ and 80 mg/cm^3^ for low and very low bone mass, as defined in the guidelines of the American College of Radiology [21]. The critical Z-score thresholds were computed based on the average vBMD for each vertebra in the control group (BMDctrl) and its standard deviation (SDctrl) with the following formulae:

Z_1_ = (120 − BMDctrl)/SDctrl

and

Z_2_ = (80 − BMDctrl)/SDctrl

Factors possibly influencing vBMD were analyzed. Criteria for the comparison were severity of scoliosis, body mass index (BMI), status of ambulation, antiepileptic drug (AED) therapy and Vitamin D supplementation. An unpaired *t*-test was used to compare the vBMD of each individual vertebra between different subgroups of the RTT patients according to criteria in Table 1.

Scoliosis was measured on ap radiographs using the Cobb method [22]. The average scoliosis angle was 79° ± 19° (range 47° to 127°). According to the severity of the scoliosis, subgroups were formed with scoliosis angle <60° (n = 5, average 53° ± 4°; range 47° to 56°) and ≥60° (n = 16; average 88° ± 15, range 61° to 127°).

The BMI calculation and range definitions were made according to the publication of Kromeyer–Hauschild et al. in 2001 [23]. Values below tenth percentile were defined as “low” (n = 9, average 13.1), while values between the tenth and nineteenth percentile or above as “normal” and “high”, respectively (n = 12, average 19.9).

We performed a simple linear regression analysis to investigate a potential correlation of vBMD with each of the parameter’s scoliosis severity, body weight, body height, and age.

Statistical analyses using post-hoc tests were performed with the statistical software package GraphPad Prism^®^ and Excel^®^ (Microsoft Corporations, Redmond, WA, USA). All data are shown as mean ± SD.

Nomenclature is based on the recommendations of the 2003 Position Development Conference (PDC) of the International Society for Clinical Densitometry (ISCD) [24] and on the working group of Rettsearch Consortium as published by Neul et al. in 2010 [1].

## 3. Results

Within five years, a total of 21 surgical corrections of neuromuscular scoliosis in Rett girls were performed. The average follow-up was 1.4 ± 1.3 years. There were no complications related to implant failure or loss of scoliosis correction. The operation-related complication rate was 14.9% (n = 3). These patients developed pleural effusions directly postoperatively, which were successfully treated with diuretic therapy and resulted in no discharge delay. There were two adverse events not related to surgery. One patient received a prolonged antibiotic therapy after developing pneumonia through aspiration, with delayed discharge. One patient acquired postoperatively an infection with COVID-19, which was additionally complicated through septic shock. After intensive treatment in the intensive care unit for two months, all supportive measures were ended with consent of the parents.

**Figure 3:**
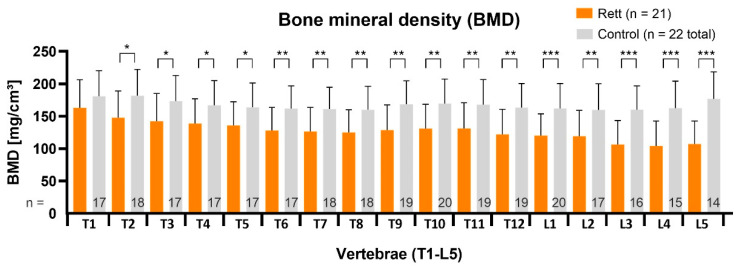
Measurements show a significant reduction of vBMD for all evaluated vertebrae, with statistical difference increasing from upper thoracic (*p* < 0.05), to thoracolumbar (*p* < 0.01) and lumbar segments (*p* < 0.001). The numbers in grey bars represent the total available vBMD values per segment in control group, which vary depending on regions examined in the available CT scans. RTT patients received whole spine scans and the number of values per segment always equals the complete group. T = thoracic vertebra; L = lumbar vertebra. Statistical significance was defined as *p* < 0.05 (*), *p* < 0.01 (**) and as *p* < 0.001 (***).

Statistical analysis showed significant results for vBMD and corresponding average BMD-Z-scores of all RTT patients (n = 21, age 13.6 ± 2.5 years, 11.0–20.6 years) in comparison to age and sex-matched healthy controls (age 13.8 ± 2.0 years, 10.6–17.9 years), as shown in Figure 3. The average Z-score of all RTT girls was −1.0 ± 0.3.

No significant differences between BMDs could be found for the severity of scoliosis, BMI or Vitamin D supplementation, or correlations using regression analysis for a relation between vBMD and severity of scoliosis, body weight, body height, and patient’s age.

### 3.1. vBMD in RTT Non-Ambulatory and Ambulatory Subgroups and Healthy Controls

**Figure 4:**
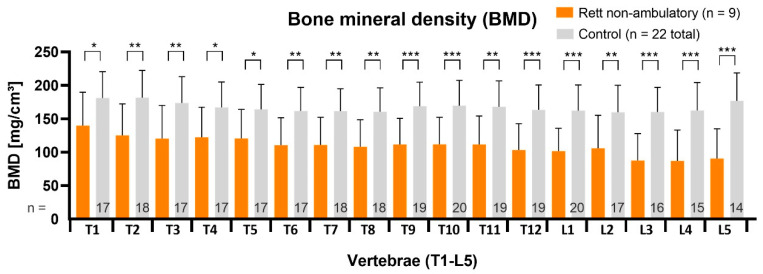
The vBMD was strongly reduced for every single vertebral segment in non-ambulatory RTT patients. T = thoracic vertebra; L = lumbar vertebra. Statistical significance was defined as *p* < 0.05 (*), *p* < 0.01 (**) and as *p* < 0.001 (***).

In the non-ambulatory RTT subgroup (n = 9, age 13.9 ± 3.2 years, 11.0–20.6 years), vBMD measurements had the most significant statistical difference, respectively, to control segments, with *p* < 0.05 in upper thoracic region, further declining in thoracolumbar direction (*p* < 0.01) and finally to demonstrate the lowest values (*p* < 0.001) in the lumbar region (Figure 4). The average BMD-Z-score was −1.5 ± 0.2.

**Figure 5:**
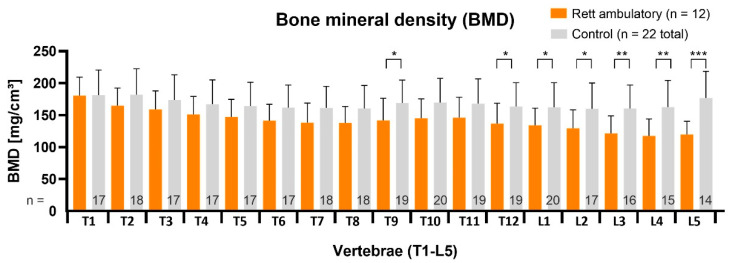
The vBMD comparison of ambulatory RTT patients (n = 12) to healthy controls. The average values show significant reduction in lumbar vertebrae. T = thoracic vertebra; L = lumbar vertebra. Statistical significance was defined as *p* < 0.05 (*), *p* < 0.01 (**) and as *p* < 0.001 (***).

The vBMD values in ambulatory RTT patients (n = 12, age 13.3 ± 1.8 years, 11.3–17.2) were altogether higher. Significantly reduced values were located only in the thoracolumbar (*p* < 0.05. *) and in the lower lumbar region (*p* < 0.01, ** and *p* < 0.001, ***) as shown in Figure 5. The average BMD Z-score was −0.7 ± 0.3.

Comparing ambulatory (n = 12) to non-ambulatory (n = 9) RTT patients, the vBMD values were significantly different at nine vertebral levels (*p* < 0.05).

### 3.2. Z-Score Evaluation in Ambulatory and Non-Ambulatory RTT Patients

**Figure 6:**
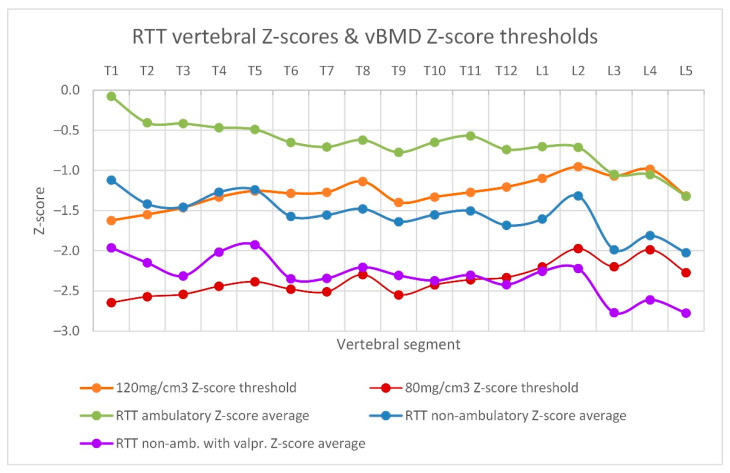
The Z-scores represent a higher fracture risk the lower they are in comparison to 120 mg/cm^3^ vBMD Z-score threshold (orange) and 80 mg/cm^3^ threshold (red). Z-scores of the ambulatory RTT subgroup remain in the normal range (n = 12, green), while those of non-ambulatory RTT girls (n = 9, blue) lie mostly below the 120 mg/cm^3^ threshold. The purple line represents the non-ambulatory RTT subgroup with valproate treatment (n = 8). Depiction of the ambulatory RTT subgroup with valproate treatment (n = 3) is omitted as it overlaps considerably with the ambulatory subgroup.

The BMD Z-scores of every segment for the ambulatory and non-ambulatory RTT subgroups in comparison to critical Z-score thresholds are depicted in Figure 6. The gradient of risk for vertebral fractures increased towards the lumbar segments, with the non-ambulatory RTT subgroup being the most affected.

### 3.3. vBMD and Valproate Treatment

**Figure 7:**
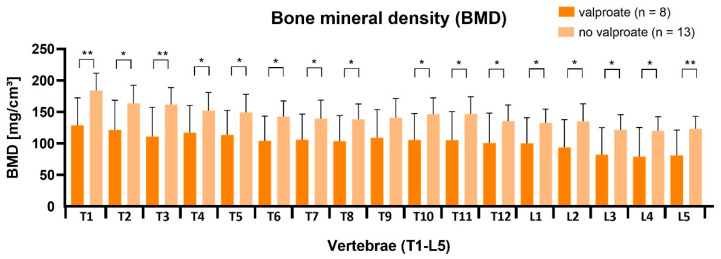
Significant vBMD reduction of almost all vertebrae in patients receiving valproate therapy, *p* < 0.05 (*) and *p* < 0.01 (**).

The RTT patients under valproate treatment had constantly significant lower vBMD values than patients without valproate or treatment with other anti-epileptic drugs (AED) (Figure 7). The subgroups consisted of ambulatory and non-ambulatory patients. This suggests a direct causative effect of valproate on BMD.

As valproate treatment causes vBMD reduction of almost all vertebrae in RTT patients, we performed an additional subgroup analysis. One subgroup consisted of ambulatory RTT girls without valproate treatment (n = 9), another of ambulatory patients with valproate treatment (n = 3), a third group of non-ambulatory RTT girls with valproate treatment (n = 5) and a fourth group (n = 4) of non-ambulatory RTT girls without valproate treatment.

The resulting average Z-score for the first and second subgroups were −0.7 ± 0.3 and −0.7 ± 0.2, respectively, both remaining in the normal range. In the third subgroup, however, the average Z-score was further reduced at −2.3 ± 0.2. The fourth group had a marginally higher average Z-score (−0.6 ± 0.3) compared to the ambulatory groups. This combined effect for the non-ambulatory, valproate treated group is shown in Figure 6.

## 4. Discussion

The RTT is an X-linked neurodevelopmental disorder which leads to stagnation and regression of cognitive and motor skills in girls from early age onwards. Patients show a variety and variability of symptoms, which are well described and revised [1]. Among them, impaired mobility or complete loss of ambulation, apraxia, gastrointestinal dysmotility, nutritional problems, and skeletal deformity are factors adversely affecting bone health. Therefore, in recent years, areal BMD through DXA examinations were the preferred method to evaluate bone mass [11,12], although obtaining accurate results may prove difficult in RTT patients because of movement disorders, contractures, and scoliosis [12].

To our knowledge, this cohort study provides the first results of spinal vBMD for RTT patients, which were proven to be more accurate than DXA based aBMD [20,25].

The results show that the state of ambulance was the factor with the most profound effect on spinal vBMD. The RTT subgroup, retaining the ability to bear full weight during ambulation, only had a slight reduction of spinal vBMD as compared to the control group and an average Z-score of −0.7 ± 0.3. Except for the lowest lumbar segments, the Z-scores of the other vertebral segments remained well above the margin corresponding to low BMD values [21]. In contrast, the non-ambulatory RTT subgroup had an average Z-score of −1.5 ± 0.2. The thoracic segmental Z-scores were well below the afore mentioned margin and declined further in lumbar segments.

One should consider that according to the ISCD official pediatric positions of 2019, Z-scores above −2 SDs in children do not eliminate the risk for a pathological fracture, especially when neuromuscular disorders are involved [10]. In 2011, a Danish study supported these findings, which showed that non-ambulatory RTT patients sustain more low-energy fractures (12 RTT females of 61 with 19 fractures) than the general healthy population (8 of 122 females with 9 fractures) [26]. However, this study and other studies [12] refer to long bone fractures, as vertebral low energy fractures are believed to remain often undetected.

The second factor to have a statistically detectable effect on vBMD was the antiepileptic treatment with valproate. This study showed that every single thoracic and lumbar vertebral segment in the valproate RTT subgroup had vBMD values which were significantly lower than their valproate naive counterparts. The average Z-score for patients without valproate treatment was −1.0 ± 0.3 and for patients with valproate was −1.7 ± 0.2. Interesting enough, valproate was the only AED to have this effect in RTT girls. This result and the fact that RTT subgroups had both ambulatory and non-ambulatory patients seem to support the assumption that valproate has a direct negative effect on BMD. Further differentiation showed that ambulatory RTT girls with and without valproate treatment had normal and almost identical average vBMD Z-scores (−0.7 ± 0.3 and −0.7 ± 0.2). In the current literature, there is limited information available regarding the effect of valproate on bone fracture risk. An Australian demographic study, through the national RTT database, demonstrated a relationship between valproate therapy and increased fracture risk in RTT [27]. Another study, in 2001, showed that AED reduced vBMD Z-scores below −1.5 SDs in children, especially in combined AED therapy [28]. Further evidence was provided by a systematic review in 2019 regarding adults, which confirms a long-term negative effect of valproate therapy on BMD only after at least three years of treatment. This could be in accordance with another study in 2015 that found no effect on BMD after two years of valproate monotherapy [29]. A mouse model study reported bone loss through modulation of estradiol and transforming growth factor beta-3 (TGF-b3) after valproate treatment [30].

Concerning the effect on non-ambulatory RTT patients with valproate treatment, the results showed a possible combined/synergic negative effect on BMD. This RTT subgroup (n = 5) had an average spinal Z-score of −2.3 ± 0.2, which corresponds to values for adult osteoporotic patients according to World Health Organization (WHO) [31]. In this study, non-ambulatory RTT girls without valproate treatment had a normal average BMD Z-score of −0.6 ± 0.3. In 2019, a study of the effects of AED on human bone cells showed that valproate can conditionally stimulate both osteoblasts and osteoclasts and influence bone turn-over rates depending on doses [32]. One could assume that the presence of osteoclasts is a prerequisite for the negative effect of AED on bone metabolism. In that case, non-ambulatory RTT girls would be even more susceptible to adverse effects of valproate, having naturally more active osteoclasts and less active osteoblasts than ambulatory girls. Whether the two factors are acting independently on BMD to cause a more severe expression of an RTT-phenotype with loss of ambulation and epilepsy, remains to be shown in future studies.

Furthermore, the relationship between scoliosis, either idiopathic or neuromuscular, in other than in RTT girls, and BMD have been examined in the literature. In adolescent idiopathic scoliosis (AIS), Cheng et al. described in 2022 a relationship between the severity of the structural curve and vBMD in spinal CT scans [33] and also found that the vertebra at the apex had the highest values compared to the other segments of the curve. Our analysis showed no relationship between the Cobb-angle and vBMD, while the values gradually reduced in craniocaudal direction, regardless of the apex location. This underlines the differences between neuromuscular scoliosis and AIS. On the other hand, the behavior of vBMD by neuromuscular scoliosis seems to also have a similar pattern in patients other than RTT. For example, our recent study on vBMD in Duchenne boys with scoliosis showed highest bone mineral density at the upper thoracic spine, which gradually declined in the direction of the lower lumbar spine [34].

Overall, this study supports the increasing evidence of the negative effect of valproate on bone mass. There is no reason why mentally impaired RTT patients, already having sustained long bone fractures, do not also suffer from silent vertebral fractures, which remain unnoticed and thus leading to chronic pain and deformity. Bisphosphonate therapy has only recently been administered to RTT patients and seems promising [35]. In non-ambulatory RRT girls receiving valproate treatment, BMD should be monitored carefully to avoid disability and pain.

Limitations of the study are clearly the relatively small numbers, especially when analyzing subgroups and their influence on vBMD. Further studies with larger cohorts will be necessary.

## 5. Conclusions

Loss of ambulance in RTT patients had a significant negative effect on bone mass with spinal vBMD reduction to osteopenia-equivalent levels, which declined even further in patients with valproate treatment. Therefore, monitoring of BMD in non-ambulatory RTT girls receiving valproate treatment could prove useful in the prevention of pathological fractures.

## Figures and Tables

**Table 1:** Main criteria for defining subgroups of RTT girls for statistical analysis of an eventual influence on vBMD. Total number of participants for each factor n = 21. Valproate was the only antiepileptic drug with relevance in the analysis and is thus included. Further non-relevant drugs were lamotrigine, levetiracetam, oxcarbazepine, perampanel sultiam, and zonisamide.

Factors	RTT Patients Criterium-Based Subgrouping	
Scoliosis angle	<60° (n = 5)	≥60° (n = 16)
Body mass index (BMI)	Low (n = 9)	Normal/high (n = 12)
Ambulation	Yes (n = 12)	No (n = 9)
Valproate treatment	Yes (n = 8)	No (n = 13)
Vitamin D supplementation	Yes (n = 12)	No (n = 9)

## Data Availability

The data presented in this study are available on request from the corresponding author.

## Abbreviations

AIS	adolescent idiopathic scoliosis
aBMD	areal bone mineral density
ap	anteroposterior
CT	computed tomography
BMD	bone mineral density
BMI	body mass index
DXA	dual-energy X-ray absorptiometry
HAZ	height adjusted Z-scores
ISCD	International Society for Clinical Densitometry
MECP2	methyl CpG binding protein 2
ROI	range of interest
RTT	Rett syndrome
SD	standard deviation
TGF-b3	transforming growth factor beta-3
vBMD	volumetric bone mineral density
UCSF	University of California, San Francisco
